# Classification of bulimic-type eating disorders: from DSM-IV to DSM-5

**DOI:** 10.1186/2050-2974-1-33

**Published:** 2013-08-20

**Authors:** Jonathan M Mond

**Affiliations:** 1 Research School of Psychology, Australian National University, Canberra ACT 0200, Australia

**Keywords:** DSM-IV, DSM-5, Bulimic-type eating disorders, Bulimia nervosa, Binge eating disorder

## Abstract

Proposed changes to the classification of bulimic-type eating disorders in the lead up to the publication of DSM-5 are reviewed. Several of the proposed changes, including according formal diagnostic status to binge eating disorder (BED), removing the separation of bulimia nervosa (BN) into purging and non-purging subtypes, and reducing the binge frequency threshold from twice per week to once per week for both BN and (BED), have considerable empirical evidence to support them and will likely have the effect of facilitating clinical practice, improving access to care, improving public and professional awareness and understanding of these disorders and stimulating the additional research needed to address at least some problematic issues. However, the omission of any reference to variants of BN characterized by subjective, but not objective, binge eating episodes, and to the undue influence of weight or shape on self-evaluation or similar cognitive criterion in relation to the diagnosis of BED, is regrettable, given their potential to inform clinical and research practice and given that there is considerable evidence to support specific reference to these distinctions. Other aspects of the proposed criteria, such as retention of behavioral indicators of impaired control associated with binge eating and the presence of marked distress regarding binge eating among the diagnostic for BED, appear anomalous in that there is little or no evidence to support their validity or clinical utility. It is hoped that these issues will be addressed in final phase of the DSM-5 development process.

## Review

Although anorexia nervosa (AN) may be the eating disorder most familiar to the public, and to medical professionals, bulimic-type eating disorders, which occur among individuals of normal or above average body weight, are far more common and affect a much broader section of the population [1-4]. These disorders include bulimia nervosa (BN) and variants of BN that fall short, in one way or another, of the criteria for diagnosis specified in current classification schemes. The most widely recognized scheme for the classification of eating disorders is the American Psychiatric Association (APA) Diagnostic and Statistical Manual of Mental Disorders (DSM), the most recent, fourth revision of which was published in 1994 (DSM-IV) [5]. Minor changes to this volume were made in text revision published in 2000 (DSM-IV-TR) [6]; however, the diagnostic criteria for most disorders, including eating disorders, were unchanged. The purpose of the current article is to review proposed changes to the classification of bulimic-type eating disorders in the upcoming, fifth revision of the DSM (DSM-5) [7].

### The status quo: classification of bulimic-type eating disorders in DSM-IV

#### BN

The current (DSM-IV/DSM-IV-TR) diagnostic criteria for BN are shown in Table 1 (top left). As can be seen, BN is characterized by “recurrent” episodes of binge eating and extreme weight-control behaviors (“inappropriate compensatory behaviors”). Binge eating entails, first, the consumption of an “objectively large” amount food in a discrete period of time and, second, a sense of loss of control over eating during the episode. Extreme weight-control behaviors include self-induced vomiting, misuse of laxatives or diuretics, extreme dietary restriction (“fasting”) and excessive exercise. “Recurrent” is defined as at least twice weekly during the past 3 months. That is, episodes of both binge eating and extreme weight control behaviors need to occur at this frequency for the DSM diagnosis of BN to be considered.

**Table 1:** Current (DSM-IV) and proposed (DSM-5) criteria for bulimic-type eating disorders: Bulimia Nervosa, binge eating disorder and eating disorders not otherwise specified (DSM-IV)/feeding or eating disorder not elsewhere classified (DSM-5)

**Current (DSM-IV) and proposed (DSM-5) diagnostic criteria for Bulimia Nervosa**	
**Current (DSM-IV)**	**Proposed (DSM-5)**
A. Recurrent episodes of binge eating. An episode of binge eating is characterized by both of the following:	A. Recurrent episodes of binge eating. An episode of binge eating is characterized by both of the following:
(1) Eating, in a discrete period of time (e.g., within any 2-hour period), an amount of food that is definitely larger than most people would eat during a similar period of time and under similar circumstances	(1) Eating, in a discrete period of time (e.g., within any 2-hour period), an amount of food that is definitely larger than most people would eat during a similar period of time under similar circumstances
(2) A sense of lack of control over eating during the episode (e.g., a feeling that one cannot stop eating or control what or how much one is eating)	(2) A sense of lack of control over eating during the episode (e.g., a feeling that one cannot stop eating or control what or how much one is eating)
B. Recurrent inappropriate compensatory behavior in order to prevent weight gain, such as self-induced vomiting; misuse of laxatives, diuretics, enemas, or other medications; fasting; or excessive exercise.	B. Recurrent inappropriate compensatory behaviors in order to prevent weight gain, such as self-induced vomiting; misuse of laxatives, diuretics, or other medications, fasting; or excessive exercise.
C. The binge eating and inappropriate compensatory behaviors both occur, on average, at least twice a week for 3 months.	C. The binge eating and inappropriate compensatory behaviors both occur, on average, at least once per week for 3 months.
D. Self-evaluation is unduly influenced by body shape and weight.	D. Self-evaluation is unduly influenced by body shape and weight.
E. The disturbance does not occur exclusively during episodes of Anorexia Nervosa.	E. The disturbance does not occur exclusively during episodes of Anorexia Nervosa.
Specify type:	
Purging Type: during the current episode of Bulimia Nervosa, the person has regularly engaged in self-induced vomiting or the misuse of laxatives, diuretics, or enemas	
Nonpurging Type: during the current episode of Bulimia Nervosa, the person has used other inappropriate compensatory behaviors, such as fasting or excessive exercise, but has not regularly engaged in self-induced vomiting or the misuse of laxatives, diuretics, or enemas	
**Current (DSM-IV) and proposed (DSM-5) diagnostic criteria for binge eating disorder**	
**Current (DSM-IV)**	**Proposed (DSM-5)**
A. Recurrent episodes of binge eating. An episode of binge eating is characterized by both of the following:	A. Recurrent episodes of binge eating. An episode of binge eating is characterized by both of the following:
1. Eating, in a discrete period of time (e.g., within any 2-hour period), an amount of food that is definitely larger than most people would eat in a similar period of time under similar circumstances	1. Eating, in a discrete period of time (e.g., within any 2-hour period), an amount of food that is definitely larger than most people would eat in a similar period of time under similar circumstances
2. A sense of lack of control over eating during the episode (for example, a feeling that one cannot stop eating or control what or how much one is eating)	2. A sense of lack of control over eating during the episode (for example, a feeling that one cannot stop eating or control what or how much one is eating)
B. The binge-eating episodes are associated with three (or more) of the following:	B. The binge-eating episodes are associated with 3 (or more) of the following:
1. Eating much more rapidly than normal	1. Eating much more rapidly than normal
2. Eating until feeling uncomfortably full	2. Eating until feeling uncomfortably full
3. Eating large amounts of food when not feeling physically hungry	3. Eating large amounts of food when not feeling physically hungry
4. Eating alone because of feeling embarrassed by how much one is eating	4. Eating alone because of feeling embarrassed by how much one is eating
5. Feeling disgusted with oneself, depressed, or very guilty after overeating	5. Feeling disgusted with oneself, depressed, or very guilty after overeating
C. Marked distress regarding binge eating is present.	C. Marked distress regarding binge eating is present.
D. The binge eating occurs, on average, at least 2 days a week for 6 months.	D. The binge eating occurs, on average, at least once a week for 3 months.
E. The binge eating is not associated with the regular use of inappropriate compensatory behaviors (e.g., purging, fasting, excessive exercise) and does not occur exclusively during the course of Anorexia Nervosa or Bulimia Nervosa.	E. The binge eating is not associated with the recurrent use of inappropriate compensatory behavior and does not occur exclusively during the course Bulimia Nervosa or Anorexia Nervosa.
**Exemplars of the DSM-IV eating disorders not otherwise specified and DSM-5 feeding or eating disorder not elsewhere classified categories**^**i**^	
**DSM-IV: Eating Disorders Not Otherwise Specified**	**DSM-5: Feeding or Eating Disorder Not Elsewhere Classified**
The Eating Disorder Not Otherwise Specified category is for disorders of eating that do not meet the criteria for any specific Eating Disorder.	**1. Atypical, mixed, or below-threshold presentations:**
	**Subthreshold Bulimia Nervosa (low frequency or limited duration)**
Examples include:	
3. All of the criteria for Bulimia Nervosa are met except that the binge eating and inappropriate compensatory mechanisms occur at a frequency of less than twice a week or for a duration of less than 3 months.	All of the criteria for Bulimia Nervosa are met, except that the binge eating and inappropriate compensatory behaviors occur, on average, less than once a week and/or for less than for fewer than for 3 months.
4. The regular use of inappropriate compensatory behavior by an individual of normal body weight after eating small amounts of food (e.g., self-induced vomiting after the consumption of two cookies).	**Subthreshold Binge Eating Disorder (low frequency or limited duration)**
	All of the criteria for Binge Eating Disorder are met, except that the binge eating occurs, on average, less than once a week and/or for fewer than for 3 months.
5. Repeatedly chewing and spitting out, but not swallowing, large amounts of food.	**2. Other specific syndromes not listed in DSM-5:**
6. Binge-eating disorder: recurrent episodes of binge eating in the absence of the regular use of inappropriate compensatory behaviors (see Appendix B in DSM-IV-TR for suggested research criteria).	**Purging Disorder**
	Recurrent purging behavior to influence weight or shape, such as self-induced vomiting, misuse of laxatives, diuretics, or other medications, in the absence of binge eating.
	**Night Eating Syndrome**
	Recurrent episodes of night eating, as manifested by eating after awakening from sleep or excessive food consumption after the evening meal. There is awareness and recall of the eating. The night eating is not better accounted for by external influences such as changes in the individual’s sleep/wake cycle or by local social norms. The night eating is associated with significant distress and/or impairment in functioning. The disordered pattern of eating is not better accounted for by Binge Eating Disorder, another psychiatric disorder, substance abuse or dependence, a general medical disorder, or an effect of medication.
	**3. Insufficient information:**
	**Other Feeding or Eating Condition Not Elsewhere Classified**
	This is a residual category for clinically significant problems meeting the definition of a Feeding or Eating Disorder but not satisfying the criteria for any other Disorder or Condition.

^i^Note. Only exemplars of the respective categories relating to bulimic-type eating disorders are shown.

A third core feature of BN is the occurrence of extreme concerns about weight or shape or, in the language of the DSM, the “undue influence of weight or shape on self-evaluation”. This feature, which is also included among the diagnostic criteria for AN, is considered by many authorities to be a *sine qua non* of eating disorder psychopathology [8,9]. The diagnosis of BN also requires that these symptoms do not occur exclusively during episodes of AN. If individuals who meet diagnostic criteria for AN have regular episodes of binge eating and/or extreme weight-control behaviors, then the diagnosis of BN is “trumped” by that of AN [10].

The final thing to note about the current (DSM-IV) classification of BN is that provision is made for subtyping of individuals with this diagnosis according to the type of extreme weight-control behaviors that they employ in attempting to compensate for their episodes of binge eating. If one or more purging behaviors, namely, self-induced vomiting and/or misuse of laxatives or diuretics, is employed, then the diagnosis of BN purging subtype is given, irrespective of whether extreme dietary restriction and/or excessive exercise is also employed. If only extreme dietary restriction and/or excessive exercise is employed, but not vomiting, laxatives or diuretics, then the diagnosis of BN non-purging subtype is given.

#### Variants of BN

In DSM-IV, variants of BN (and of AN) that, while “clinically significant”, do not meet full diagnostic criteria are designated “Eating Disorders Not Otherwise Specified” (EDNOS). The best-known variant of BN is binge eating disorder (BED), a disorder characterized by regular episodes of binge eating in the absence of the regular extreme weight-control behaviors that are characteristic of BN [9,11]. BED is also the most common variant of BN, in general population samples at least [2,4,12]. As might be expected, individuals with BED tend to be overweight, often very overweight [9,11]. This is contrast to individuals with BN, who tend to be in the normal-weight range. Further, whereas BN is far more common in women than in men, BED may be nearly as common in men as in women [1,4]. This difference reflects the fact that binge eating is common in both men and women, whereas the use of extreme weight-control behaviors, particularly purging behaviors, is uncommon in men [3].

BED was introduced, in DSM-IV, as a provisional diagnosis requiring further research and suggested diagnostic criteria were included in an Appendix [5]. As can be seen in Table 1 (middle left), these criteria include, in addition to the regular occurrence of binge eating and absence of regular extreme weight-control behaviors, the presence of 3 or more of 5 possible behavioral manifestations of binge eating and of “marked distress regarding binge eating”. As can also be seen, the operational definition of binge eating suggested for BED is identical to that for BN. For the diagnosis of BED, binge eating must occur, on average, on at least 2 days per week for a period of 6 months or more. This is in contrast to BN, for which the binge eating frequency criterion is couched in terms of the number of episodes, rather than the number of days on which episodes occur, and for which duration of 3 months is sufficient.

Other exemplars of BN-type EDNOS, that is, variants of BN other than BED, mentioned in DSM-IV include disorders that resemble BN except that the binge eating and/or compensatory/extreme weight-control behaviors occur at a frequency of less than twice per week or for a duration of less than 3 months, disorders characterized by “the regular use of inappropriate compensatory behavior by an individual of normal body weight after eating small amounts of food” and disorders characterized by “repeatedly chewing and spitting out, but not swallowing, large amounts of food” (bottom left of Table 1).

### The future: classification of bulimic-type eating disorders in DSM-5

#### BN

The proposed DSM-5 diagnostic criteria for BN are shown in the top right section of Table 1. As can be seen, the major change to the DSM-IV criteria is a revision of the frequency threshold for binge eating and extreme weight-control behaviors, so that an average frequency of once per week - as opposed to twice per week - for these behaviors is sufficient. This change is uncontroversial, since no clinically meaningful differences have been identified between individuals who otherwise meet criteria for BN and who have episodes of binge eating and compensatory behaviors at least once a week and those who otherwise meet criteria for BN and who have episodes of binge eating and compensatory behaviors at least twice per week [13].

A second, key change to the diagnostic criteria for BN, is that the subtyping of this diagnosis into purging and non-purging forms has been replaced by a single diagnosis that includes the use of both purging (self-induced vomiting, misuse of laxatives or diuretics) and non-purging (extreme dietary restriction, excessive exercise) weight-control behaviors. This latter change has been somewhat controversial in that the Eating Disorders Working Group had initially resolved to delete reference not only to the non-purging subtype of BN, but to the use of non-purging weight-control behaviors altogether. In other words, the intention was that BN would be reserved for individuals who employed purging behaviors to compensate for episodes of binge eating, whereas individuals with disorders once classified as BN non-purging subtype would, presumably, be relegated to the “Insufficient Information” section of the DSM-5 “Feeding or Eating Disorder Not Elsewhere Classified” category (bottom right of Table 1). The stated rationale for this recommendation was that:

… the non-purging subtype of BN had received relatively little attention and the available data suggested that individuals with this subtype more closely resemble individuals with binge eating disorder. In addition, precisely how to define non-purging inappropriate behaviors (e.g., fasting or excessive exercise) is unclear [7].

Subsequently, however, concerns relating to the elimination of the term “fasting or excessive exercise” from Criterion B prompted the Working Group to change its mind:

Commentators felt that these forms of inappropriate compensatory behavior, while difficult to define precisely, were observed with sufficient frequency to merit their continued inclusion in the criteria. The Work Group found this persuasive, and those terms are now proposed to be maintained in Criterion B. The text will note the lack of agreement regarding how these terms should be defined and the fact that virtually all research on Bulimia Nervosa, including its treatment, has focused on individuals who actively purge via self-induced vomiting [7].

Hence, while separation of BN into purging and non-purging subtypes will be discontinued, reference to both purging and non-purging compensatory behaviors will be retained.

This change of heart was prudent. For one thing, the fact that disorders characterized by binge eating and the use of non-purging, but not purging, weight-control behaviors, have received relatively little attention is a not a good reason to exclude these disorders from consideration. Rather, reference to these disorders needs to be retained in order to ensure that further research is in fact conducted, in the same way that the inclusion of binge eating disorder as a provisional diagnosis in DSM-IV stimulated new research addressing the prevalence and correlates of this disorder [9,11]. Otherwise the field might end up merely “studying what it defines” (or not studying what it doesn’t define) [14]. Further, eliminating reference to non-purging compensatory behaviors altogether would reinforce the impression - that subtyping gave rise to - that bulimic-type eating disorders characterized by purging behaviors are more severe than those involving non-purging behaviors when the reality is that there is little in the way of empirical evidence to support this view [1,12,15-17].

The inference that individuals who compensate for binge eating by means of extreme dietary restriction or excessive exercise, but not purging behaviors, more closely resemble individuals with binge eating disorder (than individuals who binge eat and purge) is also problematic, precisely because “how to define non-purging inappropriate behaviors (e.g., fasting or excessive exercise) is unclear” [12,17-19]. If liberal criteria for these terms (e.g. “restricting dietary intake”, “exercising hard”) are used, then the distinction between non-purging BN and BED may indeed be blurred e.g. [20]. If, on the other hand, more stringent operational definitions (e.g. “regularly going for a period of 24 hours or more without eating anything”, “exercising in a driven or compulsive manner every or nearly every day”) are used, then a clear distinction is likely to be observed e.g. [21]. The same issue arises when comparing the severity of purging and non-purging subtypes of BN [12,15,16] and in relation to variants of BN that involve extreme weight-control behaviors in the absence of binge eating (see below).

As long as there are no accepted operational definitions of “extreme dietary restriction” and “excessive exercise”, this issue will continue to be a problem for the classification of bulimic-type eating disorders. But it would be better to address this issue, for example, by testing the validity of different possible operational definitions of these terms, than to merely ignore the problem and hope that it will go away [19,22]. Population-based research will be needed to address this issue, since cases of bulimic-type eating disorders that involve non-purging, but not purging, weight-control behaviors are uncommon in clinical samples [23]. The fact that population-based research is more challenging, in terms of requiring greater investment of time and resources, than the use of convenience samples of individuals receiving specialist treatment, might explain why so little research of this kind has been conducted. In addition, the current focus of the DSM on “clinical utility” may provide an incentive for using clinical, rather than epidemiological, samples to inform revisions to classification schemes [24]. Given that the influence of the DSM extends well-beyond mental health care, to primary care practice and the community as a whole, the merits of this focus are debatable [25-27].

### Variants of BN

#### BED

The proposed DSM-5 criteria for BED are shown in the middle-right section of Table 1. As can be seen, the criteria are virtually identical to those suggested in DSM-IV, with one exception. Whereas binge eating on at least two days per week over a period of six months was required for the diagnosis of BED in DSM-IV, binge eating episodes on average at least once per week over a period of three months is required for this diagnosis in DSM-5. All of these changes - assessment of the number of binge eating episodes rather than the number of days on which binge eating occurs, a threshold for binge eating frequency of at least once, rather than at least twice, per week, and binge eating at this frequency for a period of three, rather than six, months - are uncontroversial, in that none of these factors has been found to influence the clinical significance of the disorder [13]. Further, these changes will mean that the criteria for BED are identical to those for BN in each of these respects, thereby eliminating unnecessary confusion.

Of greater consequence is the fact that in DSM-5 BED will no longer be a provisional diagnosis but, rather, a fully-fledged diagnosis, alongside AN & BN. Again this change is uncontroversial, for those in the eating disorders field at least, in that there is more than two decades of research supporting the clinical significance of eating disorders characterized by binge eating but not extreme weight-control behaviors [9,11,28,29]. Inclusion of BED as a formal diagnosis is significant, though, because it will have the effect of reducing the proportion of individuals with eating disorders who would otherwise have fallen into the DSM-5 “Feeding or Eating Disorder Not Elsewhere Classified” category [15,28]. Currently, as many as half of individuals with eating disorders receive the DSM-IV diagnosis of EDNOS and this is the case in both community and clinical samples [30,31]. Not surprisingly, concerns have been expressed about a classification scheme that relegates such a high proportion of cases to a residual category [31,32].

The inclusion of BED as a formal diagnosis also has public policy implications. Eating disorders struggle for recognition within psychiatry, let alone public health, due to the perception that these disorders are either serious but uncommon or common but trivial [1,33]. This perception has been reinforced by the low prevalence of eating disorders meeting (overly restrictive) formal diagnostic criteria. The inclusion of BED as a formal diagnosis will go some way to redressing this misconception, given the comparatively high prevalence of this disorder [2,31,34,35]. The proposed lowering of the threshold for binge eating - from twice per week to once per week - for both BN and BED, which will further reduce the proportion of cases classified as EDNOS, will consolidate change in this regard [2,31,34-36]. To the extent that reimbursement of treatment costs is contingent upon the receipt of a formal diagnosis, reducing the proportion of individuals who are classified as EDNOS should also have the effect of increasing access to specialist treatment [37].

As noted previously, BED is distinguished from other eating disorders in that it is relatively common in both men and women [2,3]. This is significant because it means that eating disorders can, or at least should, no longer be considered to be solely a problem of women [38-40]. However, as is the case with the non-purging form of BN, awareness and understanding of eating-disordered behavior in men may be poor, not only among individuals affected but among health professionals and the community as a whole [38-40]. The inclusion of BED as a formal diagnosis in DSM-5, along with reference to its epidemiology in the accompanying text, will, it is hoped, go some way to redressing this problem. More generally, there is a need to make the DSM classification of eating-disordered behavior less “female-centric” [40,41]. Some reference, in DSM-5, to the fact that this classification has been derived almost entirely from all-female samples may be helpful in promoting greater inclusion of males in future research.

Two aspects of the proposed new diagnostic criteria for BED warrant additional comment, namely, the retention of criteria B (behavioral indicators of impaired control associated with binge eating) and C (presence of marked distress regarding binge eating) and the omission of any reference to the “undue influence of weight or shape on self-evaluation”. The decision to retain criteria B and C is notable because there is virtually no empirical evidence to support the validity or clinical utility of either of these criteria [42,43]. Indeed, it has been suggested that the lack of attention given to the behavioral indicators of impaired control may reflect the fact that they are widely seen as being redundant [28]. The same could be said of criterion C [25]. There is also the issue of why these criteria were considered necessary for the diagnosis of BED but not that of BN. It is possible that concerns regarding the proliferation of diagnoses were such that there was a perceived need, on the part of the DSM-IV Eating Disorders Working Group, to take particular care to ensure the “clinical significance” of the new, provisional diagnosis of BED and that these concerns have carried over into the process of including BED as a formal diagnosis in DSM-5 [44]. However, there is considerably more evidence to support the clinical significance of BED now - irrespective of the inclusion of criteria B and C - than there was two decades ago [9,11,28,29].

The omission of any reference to the undue influence of weight or shape on self-evaluation or similar, body-image-related cognitive criterion (such as the “overvaluation of weight or shape”) from the proposed DSM-5 criteria for BED also is puzzling, given that evidence from both community and clinical samples suggests, first, that many individuals with BED or variants of BED do not have extreme concerns about weight or shape; and second, that the presence of these concerns distinguishes individuals who have very high levels of distress and disability from those who have minimal distress and disability [25,45-49]. In view of this evidence, there would seem to be a case for the inclusion of “undue influence” as a diagnostic criterion [45] or, perhaps, as a diagnostic specifier [46]. Aside from indicating disorder severity among individuals with BED and sub-threshold variants of BED, a change of this kind would bring the diagnosis of BED into line with those of both AN and BN [8,9,45].

The issue of which of the two options - diagnostic criterion or diagnostic specifier - would be preferable warrants greater consideration. As Grilo and colleagues and others have noted [46,47], inclusion of undue influence as a diagnostic criterion would likely have the effect of excluding from diagnosis some individuals who do in fact experience clinically significant levels of distress and impairment associated with their binge eating. That is, inclusion of undue influence among the diagnostic criteria for BED might result in an unacceptably high rate of “false negative diagnoses”. This may be particularly important in specialist treatment settings. On the other hand, reference to undue influence as a diagnostic specifier - but not as a criterion - might lead to the inclusion among diagnosed cases of BED of individuals who are indistinguishable, in terms of levels of eating disorder and comorbid psychopathology, from obese non-binge eaters, that is, an unacceptably high rate of “false positive diagnoses” [45,49]. This may be particularly important in primary care and other non-specialist treatment settings. There are serious adverse consequences potentially associated with both false positive (e.g. unnecessary stigma) and false negative (e.g. ineligibility for reimbursement of treatment costs) diagnoses, hence the relative priority given to minimizing these different errors needs to be carefully considered [24,50,51]. This has been done in relation to mental health problems more generally [24,50,51] but not, to the author’s knowledge, in relation to BED or other eating disorders.

In sum, the absence of any reference to undue influence in the proposed DSM-5 criteria for BED, when taken with the decision to retain criteria B and C, is difficult to fathom. However, it is important to note that the (proposed DSM-5) criteria shown in Table 1 are the most recent criteria available, rather than the final, versions of these criteria and that changes may be made before final recommendations are submitted to the APA Board of Trustees [7].

#### “Purging disorder”

In recent years, there has been considerable interest in a second variant of BN, namely, disorders characterized by the regular use of purging behaviors in the absence of regular episodes of binge eating. The term “purging disorder” has been introduced to refer to disorders of this kind and considerable evidence to support the clinical significance of these disorders has now been amassed [52,53]. Hence, purging disorder is included as an exemplar of the DSM-5 “Feeding or Eating Disorder Not Elsewhere Classified” category (bottom right of Table 1). It is described as being characterized by “Recurrent purging behavior to influence weight or shape, such as self-induced vomiting, misuse of laxatives, diuretics, or other medications, in the absence of binge eating” [53].

As I have argued elsewhere [1,54], there are at least two conceptual problems with the notion of a “purging disorder”. First, individuals who report the regular use of purging behaviors in the absence of binge eating often also report regular episodes of subjective binge eating, that is, episodes of perceived over-eating in which a loss of control is experienced but the amount of food consumed is not objectively large, and the available evidence suggests that it is the combination of subjective binge eating and purging behaviors, rather than the occurrence of purging per se, that accounts for the distress and disability associated with these conditions [1,14,54,55]. There is nothing surprising about this latter finding. Evidence from epidemiological studies has consistently shown that, among individuals with bulimic-type eating disorders, the experience of loss of control over eating is a better predictor of distress and disability than the amount of food consumed [1,14,55-60]. This evidence calls into question the validity of the DSM definition of binge eating, which requires not only a loss of control over eating but consumption of an objectively large amount of food.

Whereas “the regular use of inappropriate compensatory behaviors by an individual of normal body weight after eating small amounts of food” is included as an exemplar of EDNOS in DSM-IV, in DSM-5 this terminology is omitted in favor of “purging disorder”. The concern with this change is that the focus on purging, rather than the combination of subjective binge eating and purging, may divert attention away from the need for further research addressing the validity of the current DSM definition of binge eating [15,22,55-58]. Since bulimic-type eating disorders characterized by subjective, but not objective, binge eating are relatively uncommon in clinical samples, community-based research will be needed to address this issue and, as also noted above, community-based research may not be a priority for the architects of DSM-5 [24]. On the other hand, the fact that disorders of this kind are uncommon in clinical samples may reflect, in part, the tendency to study - and treat - what we define [14,23].

A concern relating to the inclusion, or at least mention, in the DSM of variants of BN characterized by subjective binge eating is that the assessment of subjective binge eating has been found to be unreliable, far more unreliable than that of objective binge eating [61-63]. This is hardly surprising, given the difficulty of determining whether or not a loss of control over eating is experienced when the amount of food consumed is not unusually large. Further, any change to classification schemes in which eating disorders characterized by SBEs, but not OBEs, are given formal recognition raises vexed conceptual issues, including the very definition of “eating disorder” [64]. As with bulimic-type disorders characterized by non-purging, but not purging, compensatory behaviors, however, simply ignoring the issue is not a good option. Reference to variants of BN characterized by subjective, but not objective, binge eating, in DSM-5 would serve the dual purposes of alerting clinicians - primary care and other non-specialist practitioners in particular - to the occurrence of these disorders while also alerting researchers of the need to address issues of assessment [15,56-58]. It might also be noted that, in adults at least, virtually nothing is known about the prevalence and correlates of variants of BED characterized by SBEs, that is, conditions that involve neither recurrent OBEs nor recurrent extreme weight-control behaviors. Hence, at present, a definition of binge eating solely in terms of loss of control over eating could be considered only in relation to the diagnosis of BN.

A second problem with “purging disorder” is that the use of this term may be taken to infer that variants of BN characterized by subjective, but not objective, binge eating, are worthy of attention if, and only if, they entail the use of purging behaviors. Admittedly, research addressing the clinical significance of disorders characterized by recurrent episodes of subjective binge eating and the recurrent use of non-purging - but not purging - weight-control behaviors is limited. No doubt the lack of any agreed-upon operational definitions of extreme dietary restriction and excessive exercise is a factor in this [12,17,19]. However, findings from at least one population-based study suggested that the combination of binge eating and extreme weight-control behaviors is associated with marked impairment in psycho-social functioning and that this is the case whether for both objective or subjective binge eating and for both purging and non-purging weight-control behaviors [1].

Taken together, the considerations outlined above suggest that the term “compensatory disorder” [1,65] may be preferable to “purging disorder” when referring to disorders characterized by the regular occurrence of extreme weight-control behaviors in the absence of regular (objective) binge eating. A third option that has occasionally appeared in the literature, namely, “subjective bulimia nervosa”, may be less desirable because the use of this term may be taken to infer a condition that is less severe or deserving of clinical attention [14,66].

#### Night eating syndrome

Some comment is warranted concerning so-called “night eating syndrome” (NES), given the intention to include this “disorder”, alongside “purging disorder”, as an exemplar of the DSM-5 “Feeding or Eating Disorder Not Elsewhere Classified” category. As the name suggests, NES is characterized by recurrent episodes of night eating, as manifested by excessive food consumption at night (“evening hyperphagia”) and/or episodes of eating after awakening from sleep (“nocturnal eating”) [67]. Negligible food intake at breakfast (“morning anorexia”) has also been included as a criterion in some studies [67]. The first scientific study of this behavior, based on a case series study of obese individuals receiving specialist treatment, was published in 1955, i.e. long before the inclusion of either AN or BN in the DSM [68,69]. However, despite this long history, NES has not previously been mentioned in the DSM classification of eating disorders. Hence the proposal to do so in DSM-5 is significant.

The decision to include NES as an exemplar of the DSM-5 Feeding or Eating Disorder Not Elsewhere Classified category is also somewhat surprising, however, given that a recent, comprehensive review of the literature found concluded that there was little evidence to support either the validity or clinical utility of this construct as a potential eating disorder diagnosis [70]. Problems with the existing evidence identified in that review included the lack of agreed upon definitions of and operational criteria for the core features of NES, the reliance on convenience samples of obesity patients in research seeking to elucidate the characteristics and correlates of the behaviors concerned, and, in turn, the lack of any compelling evidence for the delineation of NES from other forms of disordered eating or from normalcy [70]. On the other hand, if specific reference to NES in DSM-5 has the effect of stimulating the additional research needed to address these issues, then the decision to include such reference may be justified.

The decision to include sub-threshold forms of the proposed new BN and BED diagnoses, namely, variants of these disorders characterized by binge eating that occurs less than weekly or for a duration of less than 3 months, as exemplars of the DSM-5 Feeding or Eating Disorder Not Elsewhere Classified category might also be questioned, given that evidence bearing on the clinical significance of these conditions is currently limited [13]. On the other hand, findings from those few studies that have considered bulimic-type eating disorders involving less-than-weekly binge eating suggest that these conditions may be associated with levels of distress and disability comparable to those observed in disorders involving more frequent binge episodes [13]. Hence, it could be argued that further research addressing the optimal diagnostic threshold for binge frequency is warranted and that specific reference to these new sub-threshold variants of BN and BED within the DSM-5 Feeding or Eating Disorder Not Elsewhere Classified category will provide an incentive in this regard. In the meantime, severity of binge eating might be specified as a dimensional rating, so that clinicians and/or researchers would be required to rate the severity of binge eating according to its frequency and, perhaps, an additional, dimensional rating of distress or disability associated with binge eating behaviors [13,56].

Finally, readers may observe that reference to variants of BN characterized by “repeatedly chewing and spitting out, but not swallowing, large amounts of food” as an exemplar of the EDNOS category has been abandoned in DSM-5. This change presumably reflects the perception that these behaviors, which have been observed among individuals with both AN-type and BN-type disorders, are no longer of sufficient importance, in terms of their prevalence and/or clinical significance, to warrant specific mention, although there appears to be little in the way of empirical evidence to support such an assumption [71,72].

## Conclusions

Proposed changes to the DSM diagnostic criteria for bulimic-type eating disorders in the lead up to the publication of DSM-5 will go some way to facilitating clinical practice, improving access to care, improving public and professional awareness and understanding of these disorders and stimulating the additional research needed to address at least some problematic issues. However, the omission of any reference to variants of BN characterized by subjective, but not objective, binge eating episodes, and to the undue influence of weight or shape on self-evaluation or similar cognitive criterion in relation to the proposed criteria for BED, is regrettable and other aspects of the proposed criteria, such as retention of behavioral indicators of impaired control associated with binge eating and the presence of marked distress regarding binge eating among the diagnostic criteria for BED, appear anomalous. It is possible that these issues will be addressed in the final phase of the DSM-5 development process.

### Postscript

Since this manuscript was reviewed, DSM-5 has been published [73]. There were no changes to the wording of the diagnostic criteria for BN or BED as shown in Table 1. For both diagnoses, these criteria have been supplemented with two sets of specifiers indicating, respectively, the occurrence/state of remission (partial, full) and the current level of severity (mild, moderate, severe, extreme). Also for both diagnoses, operational definitions of each category of remission (in terms of some, or all, of the diagnostic criteria no longer being met for a sustained period of time after full criteria were previously met) and severity (in terms of the weekly frequency of inappropriate compensatory behaviours for BN and weekly frequency of binge eating for BED) are suggested. The inclusion of these specifiers is consistent with the stated intention of the DSM-5 Taskforce to improve clinical utility through the addition of specifiers or dimensions that serve to communicate clinically salient features of a disorder [24].

Concerning the proposed DSM-5 category of “Feeding or Eating Disorder Not Elsewhere Classified”, this term has been replaced by a new term, namely, “Other Specified Feeding or Eating Disorder”, that subsumes each of the four specific exemplars of this broad category (sub-threshold BN, sub-threshold BED, Purging Disorder and Night Eating Syndrome) shown in Table 1. Accordingly, use of the sub-headings “Atypical, mixed, or below-threshold presentations” and “Other specific syndromes not listed in DSM-5” has been omitted. In addition, the term “Other Feeding or Eating Condition Not Elsewhere Classified” has been replaced by “Unspecified Feeding or Eating Disorder” and the latter is included as a separate, fourth category of disorder, as opposed to being subsumed within the “Other Specified Feeding or Eating Disorder” category. The previously included “Insufficient Information” sub-heading has also been deleted accordingly. These changes presumably reflect a consolidation of efforts to preclude trivialisation of disorders that do not meet formal diagnostic criteria for AN, BN or BED [1,74].

## Competing interests

The author declares that he has no competing interests.
